# The age-regulated zinc finger factor ZNF367 is a new modulator of neuroblast proliferation during embryonic neurogenesis

**DOI:** 10.1038/s41598-018-30302-2

**Published:** 2018-08-07

**Authors:** Valentina Naef, Sara Monticelli, Debora Corsinovi, Maria Teresa Mazzetto, Alessandro Cellerino, Michela Ori

**Affiliations:** 1 Unità di Biologia Cellulare e dello Sviluppo, Dipartimento di Biologia, Università di Pisa, Pisa, I-56127 Italy; 2 Scuola Normale Superiore, Laboratory of Biology (Bio@SNS), Pisa, I-56124 Italy; 3 Leibniz-Institut für Alternsforschung, Fritz-Lipmann Institut Jena, Jena, D-07745 Germany

## Abstract

Global population aging is one of the major social and economic challenges of contemporary society. During aging the progressive decline in physiological functions has serious consequences for all organs including brain. The age-related incidence of neurodegenerative diseases coincides with the sharp decline of the amount and functionality of adult neural stem cells. Recently, we identified a short list of brain age-regulated genes by means of next-generation sequencing. Among them *znf367* codes for a transcription factor that represents a central node in gene co-regulation networks during aging, but whose function in the central nervous system (CNS), is completely unknown. As proof of concept, we analysed the role of *znf367* during *Xenopus laevis* neurogenesis. By means of a gene loss of function approach limited to the CNS, we suggested that *znf367* might act as a key controller of the neuroblast cell cycle, particularly in the progression of mitosis and spindle checkpoint. A candidate gene approach based on a weighted-gene co-expression network analysis, revealed *fancd2* and *ska3* as possible targets of znf367. The age-related decline of *znf36*7 correlated well with its role during embryonic neurogenesis, opening new lines of investigation also in adult neurogenesis to improved maintenance and even repair of neuronal function.

## Introduction

The age-related incidence of many brain diseases coincides with a reduced adult neurogenic potential. The regenerative capability and the amount of adult neural stem cells (aNSCs) decline with age, contributing to the reduced functionality of the aged brain^1^. Despite the great interest in age related diseases, the molecular factors responsible for age-dependent decay of aNSCs function and the transition between stemness and differentiating properties of these precursors are almost unknown. Recently, we identified a set of evolutionarily-conserved genes expressed in aNSCs and age-regulated by RNA-seq analysis in the short-lived fish *Nothobranchius furzeri*, a well-established animal model in aging studies^2^. Among them, zinc finger protein 367 (znf367) was suggested to occupy a central position in a regulatory network controlling cell cycle progression and DNA replication. We found that *znf367* is expressed in the adult brain of *N*. *furzeri*, where its RNA level decreases with age, and in neuroblast and retinoblast of developing *Zebrafish* embryos^2^. Znf367 belongs to the Zinc finger (ZNF) transcription factors family that represents a large class of proteins that are encoded by 2% of human genes^3,4^. Their functions include DNA recognition, RNA packaging, transcriptional activation, regulation of apoptosis, protein folding and assembly, and lipid binding^5^. Zinc finger proteins have an evolutionarily conserved structure and the ones containing the Cys_2_-His_2_ motif, constitute the largest family^6^. The function of the majority of ZNF genes is largely unknown, but some of them play a critical role in the development and differentiation of the nervous system. For instance, the Kruppel-like zinc finger transcription factor *Zic* has multiple roles in the regulation of proliferation and differentiation of neural progenitors in the medial forebrain and cerebellum^7^. The *Ikaros* family of transcription factors is characterized by two sets of highly conserved Cys_2_His_2-_type zinc finger motif and is involved in the maturation and differentiation of striatal medium spiny neurons^8^. *Znf367* gene (also known as *ZFF29 or ZFP367*) has been initially isolated in human fetal liver erythroid cells^9^. In the human genome, this gene is on chr 9q and two alterative mRNA splicing products were identified and designated ZFF29a and ZFF29b. They both code for nuclear proteins, but only ZFF29b seems to act as an activator factor of erythroid gene promoters^9^. In Human SW13 adrenocortical carcinoma cell line, *znf367* is overexpressed and in this cell line *Znf367* downregulation caused an increase of cellular proliferation, invasion and migration^10^. Furthermore, *znf367* was also identified as a potential tissue-specific biomarker correlated with breast cancer where its expression level is dysregulated influencing cell proliferation, differentiation and metastatic processes^11^. To our knowledge, there are no data available regarding the putative role of *znf367* in the Central Nervous System (CNS) during embryonic and adult neurogenesis. A very recent research paper analyzed the transcriptome of different pools of aNSC that comprise quiescent and activated neural stem cells in the mouse sub-ventricular zone^12^. Interestingly in the Supplementary materials (Table S7), the authors compared the trascriptome of young (3–4 month-old) quiescent neural stem cells to the one obtained from old (19–22 month-old) quiescent neural stem cells and *znf367* (*zfp367*) emerged among the genes significantly down regulated in the old mice^12^. Despite this data confirmed that, even in mammals, *znf367* is an age-regulated gene in the adult brain, its function in the CNS remained unknown. To shed light on the *znf367* role in vertebrates CNS, we analyzed its function during *Xenopus laevis* neurogenesis. The clawed frog *Xenopus* is the favorite animal model to perform functional screening of genes. In *Xenopus*, it is possible to microinject mRNAs or morpholino oligos in just one side of the early cleaving embryo and compare, in each embryo, the manipulated side of the embryo with its wild-type counterpart that represents a perfect internal control. This unique vertebrate model also provide the possibility, to rapidly perform gene loss of function experiments in a tissue specific manner thanks to the well-defined fate map of each blastomere of the early cleaving embryo. This allowed us to target specific *znf367* morpholinos to the central nervous system without interfering with the normal development of the other tissues. In this paper, we show that *znf367* is expressed in the developing CNS in *Xenopus* and it could have a key role in primary neurogenesis, regulating the neuroblast progression of mitosis. These finding, together with the *znf367* gene expression decline observed during CNS aging, lay the groundwork for future studies aimed to unveil znf367 role in adult neurogenesis and CNS aging.

## Results

### Embryonic expression analysis of *znf367*

**Figure 1:**
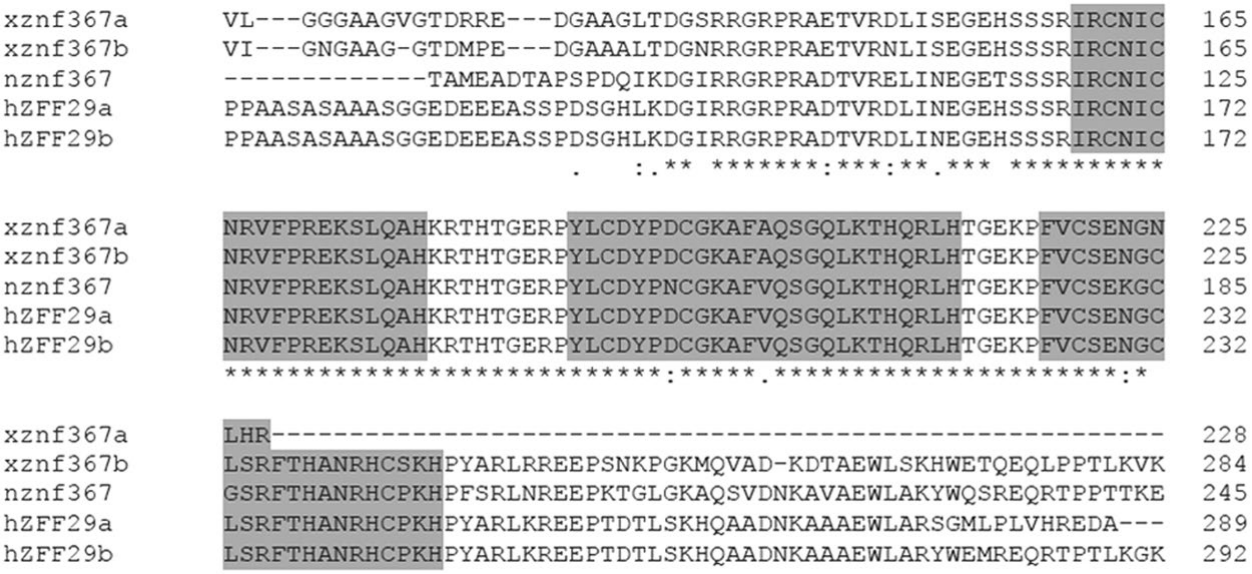
Multiple sequence alignments of znf367 amino acid sequences. The gray boxes highlighted the conserved zinc-fingers binding domains of znf367 from *X*. *laevis* (both splicing variants znf367a and znf367b), human (both splicing variants ZFF29a and ZFF29b) and *N*. *furzeri* (n) obtained using Clustal Omega.

**Figure 2:**
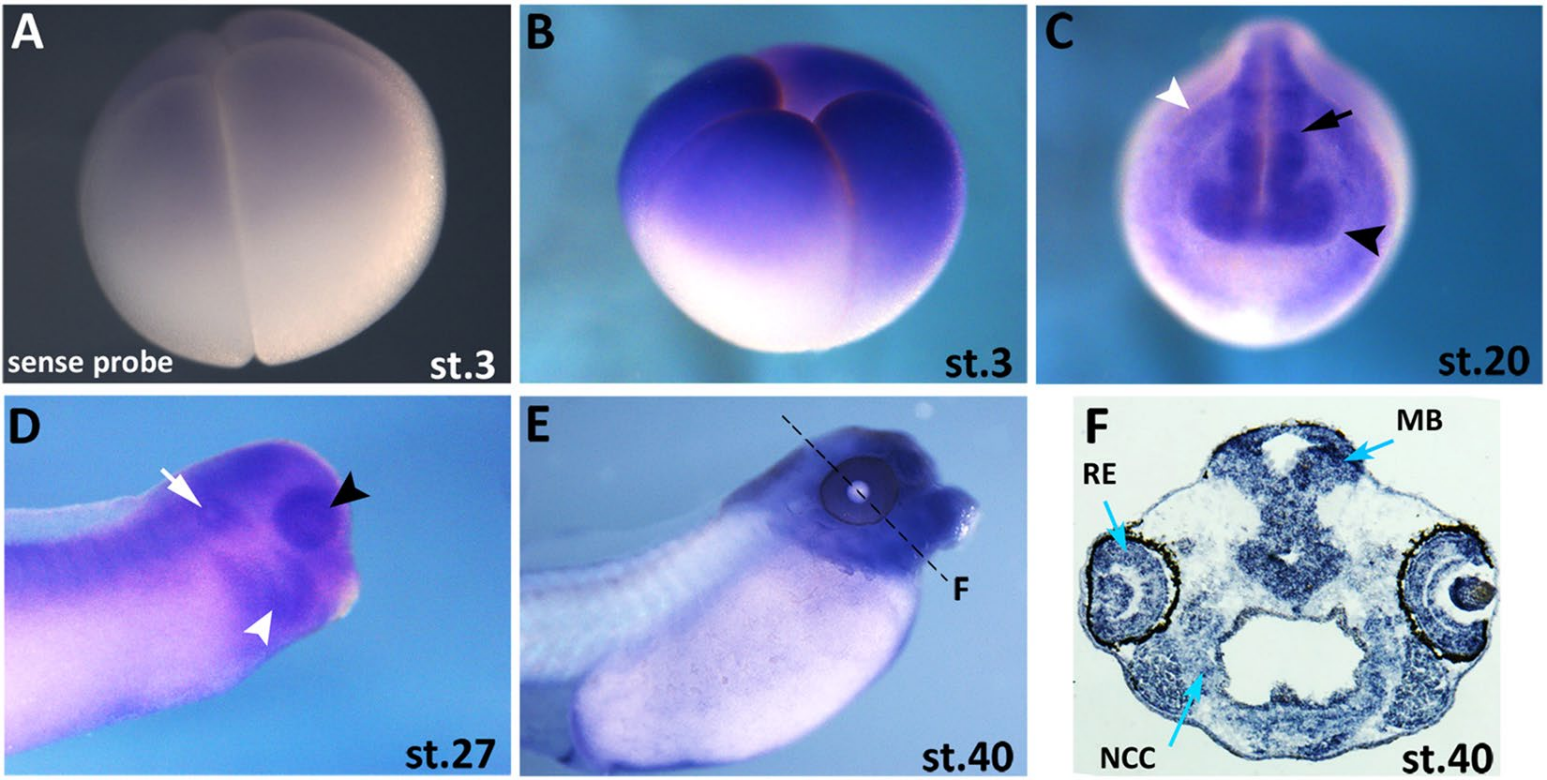
*Znf367* gene expression pattern during *Xenopus laevis* development. (**A**,**B)** *Znf367* expression at blastula stage (stage 3) using sense control probe (A) and antisense probe (B). **(C**,**D)** At neurula (C) and at tadpole stages (D) *znf367* is expressed in the neural tube (black arrow), the developing eye (black arrowhead), the neural crest cells (white arrowhead) in the otic vesicle (white arrow) and in the most anterior regions of the nervous system. **(E**,**F)** Stage 40 embryo is shown in lateral view (E) and in a transversal section at the level of the hindbrain (F). MB, midbrain; NCC, skeletogenic neural crest cells; RE, retina.

To verify the evolutionary conservation of znf367 sequence in vertebrates we performed an *in silico* analysis of the amino acid sequences of ZNF367 in *Homo sapiens* (both splicing variants: ZFF29a and ZFF29b), *Nothobranchius furzeri* and *Xenopus laevis* (both splicing variants: *znf367a* and *znf367b*). This approach revealed a high conservation of znf367 with a 66% of identity between the human and *Xenopus* amino acid sequence that reached 98% at the level of the zinc finger domains (Fig. 1) suggesting a conserved putative znf367 function in vertebrates, from fish to tetrapod and primates. To analyze the spatio-temporal gene expression pattern of *znf367*, whole mount *in situ* hybridization (WISH) was performed on *Xenopus* embryos at different stages. Z*nf367* is expressed maternally in the animal pole in *Xenopus* embryos at blastula stage (Fig. 2A,B) when compared to sense control probe treated siblings (Fig. 2A). At neurula stage *znf367* is expressed in the neural tube, in the eye fields, in the pre-placodal territory and in the neural crest cells (NCC) (Fig. 2C). At the tadpole stage, *znf367* is widely expressed in the central nervous system, in the eye, in the otic vesicle and in the NCC migrated in the branchial pouches (Fig. 2D). At larval stages of development, *znf367* is widely expressed in the CNS as shown in transverse sections (Fig. 2E,F).

### *Znf367* Knockdown inhibits neuronal differentiation in *Xenopus laevis* embryos

**Figure 3:**
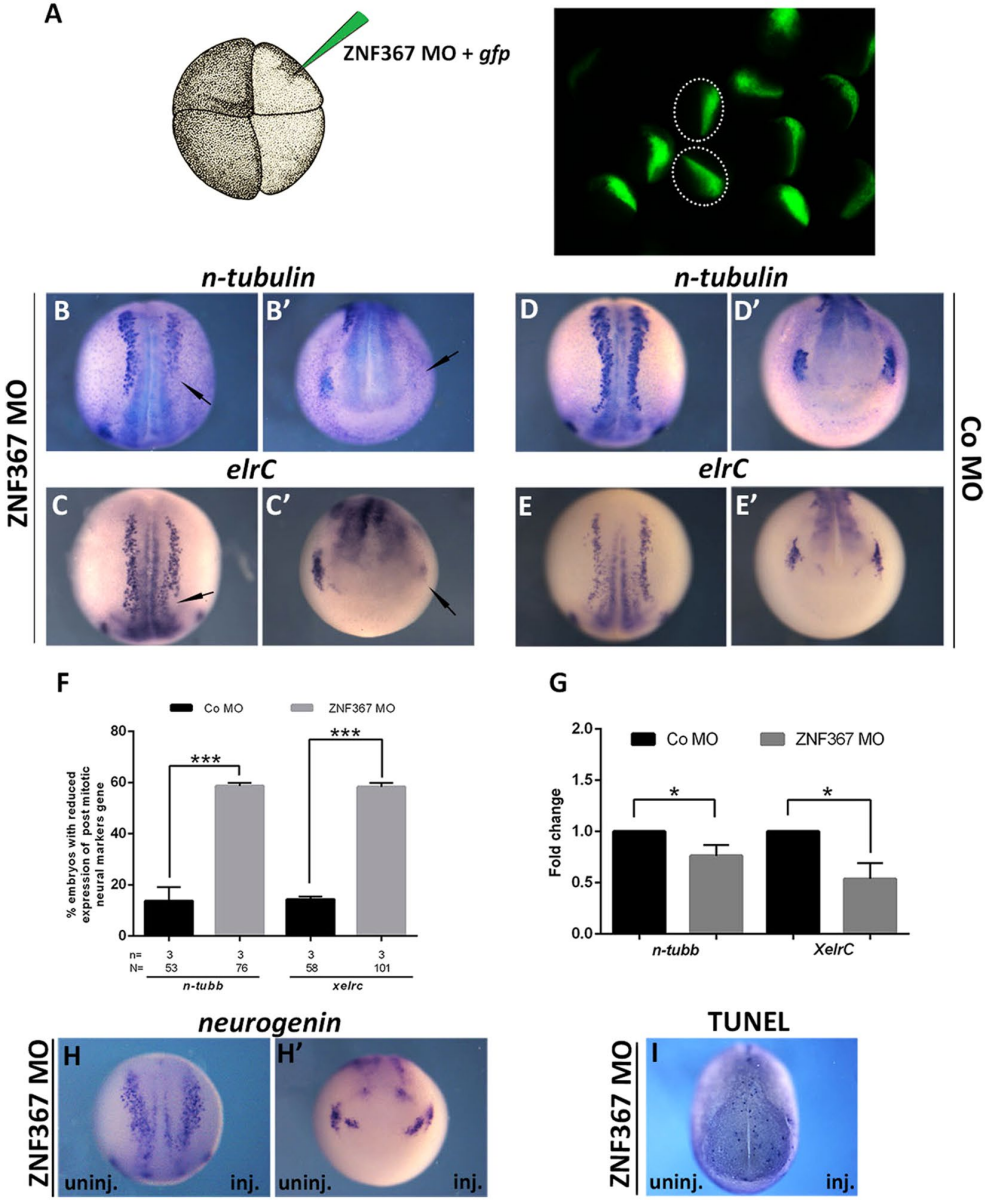
Loss of *znf367* function interferes with the expression of neuronal differentiation markers. (**A**) Embryos injected with *gfp (*250 pg) and either ZNF367-MO or Control morpholino (Co-MO) (9 ng) at one dorsal blastomere at the four-cells stage showed fluorescence only in the neural plate at neurula stage (st. 18). **(B–**E’**)** mRNA distribution of *N-tubulin* and *elrC* in *znf367* morphants and controls. (**B**,**C**) dorsal view and (B’,C’) frontal view of neurula morphants showing a clear loss of differentiation markers expression in primary neurons (arrows). (**D**,**E**) dorsal view and (D’,E’) frontal view of neurula embryos injected with Co-MO. **(F)** Quantification of the data in A and B. **(G)** qRT-PCR analysis. The levels of expression for the indicated mRNAs were evaluated for Co-MO or ZNF367-MO populations by qRT-PCR, and normalized to that of the housekeeping gene, *glyceraldehyde 3-phosphate dehydrogenase* (*gapdh*) expression. The mean of the Control-MO was set to 1. For each gene, three independent RNA samples from morphants and controls were analysed. **(H–**H’**)** mRNA distribution of *neurogenin* in *znf367* morphants. **(I)** TUNEL staining. ZNF367-MO injection did not lead to an increase of TUNEL positive cells compared to the un-injected side. Abbreviations: n, number of independent experiments; N, number of evaluate embryos in total; Error bars indicate standard error of the means (s.e.m); *p ≤ 0,05; ***p ≤ 0,001. P-value were calculated by Student’s t-test.

To investigate the *znf367* function during neurogenesis in *Xenopus*, we performed knockdown experiments using a specific antisense oligonucleotide morpholino designed to block the translation of the endogenous mRNA (ZNF367-MO). For all the experiments described here, injections were performed unilaterally into one dorso-animal blastomere at the four-cell stage to target the neural tissue. The un-injected side served as an internal control and the co-injection with *gfp* (250 pg) RNA was used to screen for correctly injected embryos (Fig. 3A). The standard Gene Tools Control-morpholino (co-MO) was used to control for non-specific embryo responses. At neurula stage (st.18), *znf367* morphants showed a strong reduction of cells expressing the markers of post-mitotic neurons *N-tubulin* and *elrC* (also known as *HuC)* in the injected side of the embryos compared to the control side and the co-MO injected embryos (Fig. 3B–E’). These data were also confirmed by qRT-PCR analysis that showed a significant reduction of both neuronal markers in *znf367* morphants (Fig. 3F,G). Interestingly, the injection of ZNF367-MO did not affect the expression of *ngnr1*, a proneural marker necessary for the specification of primary neurons^13^ (N = 53) (Fig. 3H–H’), suggesting a role for *znf367* during neuronal differentiation, but not in neuronal specification. The lack of differentiated primary neurons in *znf367* morphants could be due to an increase in cell apoptosis during the differentiation process. To evaluate this aspect, we performed a TUNEL assay in *znf367* morphants at the neurula stage and did not detect a significant increase in TUNEL positive cells between the *znf367* injected side and the un-injected control side of each analyzed embryo (Fig. 3I).

### *Znf367* knockdown increases proliferation markers in *Xenopus laevis* embryos

**Figure 4:**
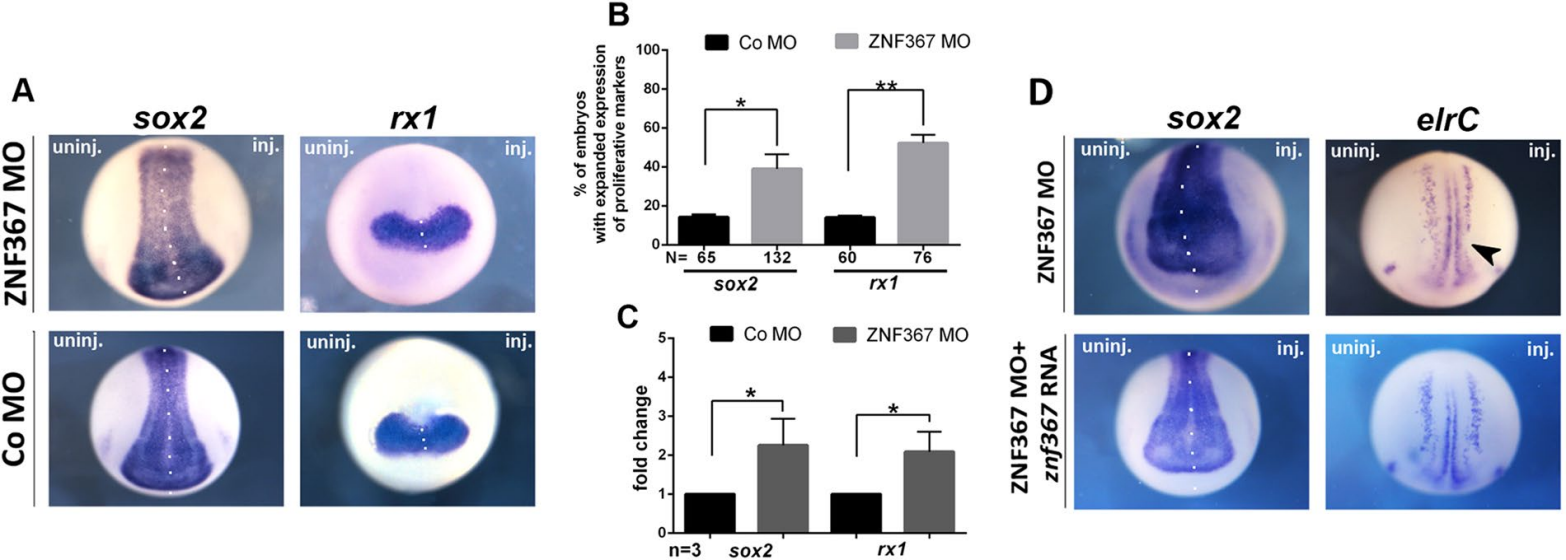
*znf367* morphants analysis and control rescue experiments. (**A)** mRNA distribution of *sox2* and *rx1* in *znf367* morphants and controls. **(B)** Statistical analysis of the data in A. (**C**) The levels of expression for the indicated mRNAs were evaluated for Co-MO or ZNF367-MO populations by qRT-PCR, and normalized to that of the housekeeping gene, *glyceraldehyde 3-phosphate dehydrogenase* (*gapdh*) expression, and the mean of the Control-MO was set to 1. For each gene, three independent RNA samples from morphants and controls were analysed. **(D)** The morphants phenotype can be rescued by the co-injection of morpholino plus full length *Xenopus znf367* mRNA as shown by the recovered expression of *sox2* and *elrC*. Abbreviations: n, number of independent experiments; N, number of evaluated embryos in total; Error bars indicate standard error of the means (s.e.m); *p ≤ 0,05; **p ≤ 0,01. P-value were calculated by Student’s t-test.

**Figure 5:**
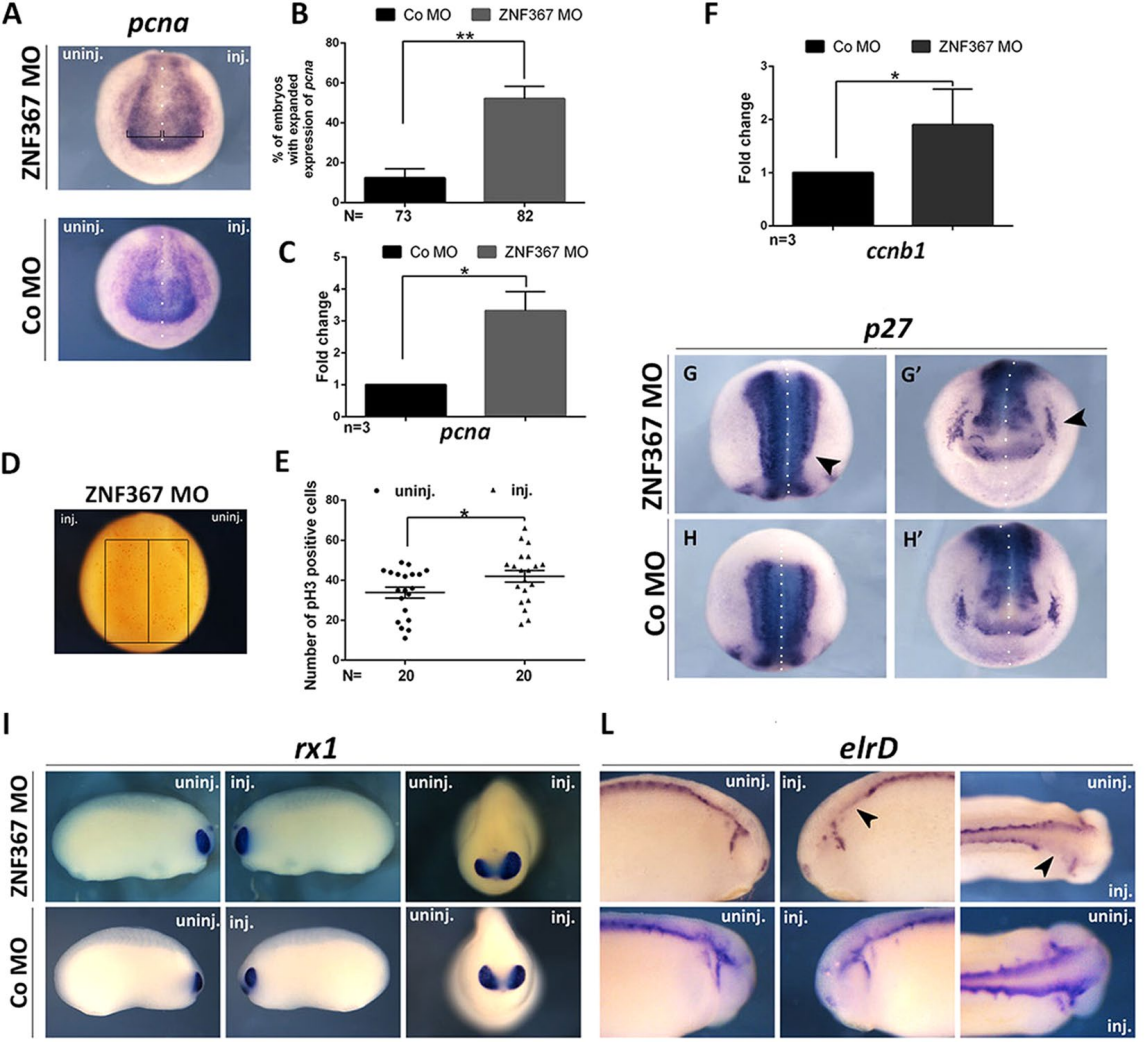
*znf367* morphants analysis for proliferating and differentiating precursors (**A)** mRNA distribution of *pcna* in *znf367* morphant and control. **(B)** Statistical analysis of the data in A. **(C)** RT-PCR analysis revealed a significant increase of *pcna*. **(D,E)** *Znf367* depletion leads to a significant reduction of proliferating cells compared to the un-injected side. pH3 positive cells were counted in the areas defined by the black rectangles. Statistical evaluation of the data is shown (E). **(F)** RT-PCR analysis of *cyclinB1* (*ccnb1*). The levels of expression for the indicated mRNAs were evaluated for Co-MO or ZNF367-MO embryos by qRT-PCR in triplicate, and normalized to *glyceraldehyde 3-phosphate dehydrogenase* (*gapdh*) expression. **(G–**G’) *p27* is down regulated at stage 18 in *znf367* morphants (arrowheads). (**G**) dorsal view; (G’) frontal view. **(H–**H’). *p27* expression in control embryo, (H) dorsal view; (H’) frontal view. **(I–L)** *rx1* and *elrD* expression at tailbud stages confirmed the phenotype observed at neurula stages: increased expression of *rx1* and downregulation of a neuronal differentiation marker (*elrD*) (arrowheads). Abbreviations: n, number of independent experiments; N, number of evaluated embryos in total; Error bars indicate standard error of the means (s.e.m); *p ≤ 0,05; **p ≤ 0,01. P-value were calculated by Student’s T-test.

To determine whether the observed loss of post-mitotic neurons in *znf367* morphants was the consequence of impairment in the maintenance of the neuronal progenitors pool, we visualized cells expressing the stemness genes *sox2* and *rx1*, in the injected embryos. *Sox2* and *rx1* are involved in maintaining neuroblast and retinoblast as cycling precursors in the neural plate^14,15^. The expression domains of *sox2* and *rx1* were expanded on the ZNF367-MO injected side of the embryo as compared to either the un-injected and Co-MO injected sides (Fig. 4A,B) indicating a larger population of progenitors. These data were confirmed by qRT-PCR analysis that showed a significant increase of both mRNAs in *znf367* morphants (Fig. 4C). On the base of these results, we can suggest that the *znf367* knockdown enhances self-renewal of progenitors at the expense of differentiation. For testing the specificity of the ZNF367-MO to induce this phenotype, we performed functional rescue experiments by co-injecting 9 ng ZNF367 MO together with 500 pg full-length *Xenopus znf367* mRNA. We observed a restoration of the phenotype of the injected embryos visualized by the *sox2* and *elrC* markers at neurula stage (30% of rescue for *sox2* N = 114; 25% of rescue for *elrC* N = 100) (Fig. 4D). To further verify whether *znf367* downregulation could alter the regulation of neuroblast proliferation, we also examined the mRNA expression of *pcna (proliferating cell nuclear antigen)* and we directly counted mitotically active cells marked by the anti-phosphorylated H3 (p-H3) antibody. *Znf367* morphants showed an increased *pcna* mRNA expression both in WISH (Fig. 5A,B) and qRT-PCR experiments (Fig. 5C). The p-H3 staining showed a significant increase in mitotic cells number upon ZNF367-MO injection as compared to the control side (Fig. 5D,E). Given that a larger pool of neuroblasts did not correspond to an increased number of differentiated cells in the absence of apoptotic cell death, it is tempting to speculate that *znf367* could be required to exit the M phase or control the mitotic checkpoint that precedes the anaphase. To test this hypothesis, we first evaluated the relative expression of *cyclin B1* that is expressed predominantly during M phase of the cell cycle^16^, by qRT-PCR analysis of *znf367* morphants. This experiment revealed a significant increase of *cyclin B1* expression in znf367 morphants (Fig. 5F) indicating again that *znf367* deficient neuroblasts could enter M phase, but they could not correctly exit mitosis and differentiate. The differentiation of neuronal progenitors requires the withdrawal from the cell cycle, driven by cell cycle inhibitors, such as *pak3 (p21)* and *p27*^17,18^. Concomitantly with the increase in mitotically active cells, a mild loss of *p27* expression (phenotype 55%, N = 93) was observed in neurula morphants indicating that *znf367* depleted neuroblasts are unable to exit the cell cycle (Fig. 5G–H’). These data led us to hypothesize that *znf367* could be involved in the cell cycle exit and/or for the initiation of maintenance of a differentiated state. Finally, we examined morphants at the tailbud stage by performing WISH using *rx1* and *elrD* (also known as *HuD*). *ElrD* labels post-mitotic neurons in the neural tube and the developing cranial ganglia^19^. As stated above, *znf367* knockdown, but not control MO, caused an increase in *rx1* gene expression (52%, N = 72) (Fig. 5I) while inhibiting neuronal differentiation, thus affecting the expression of *elrD* (54% N = 64) (Fig. 5L). These data showed that the effects of depletion *znf367* are not recovered even in the late phases of primary neurogenesis.

### Identification of putative Z*nf367* targets: a candidate gene approach

**Figure 6:**
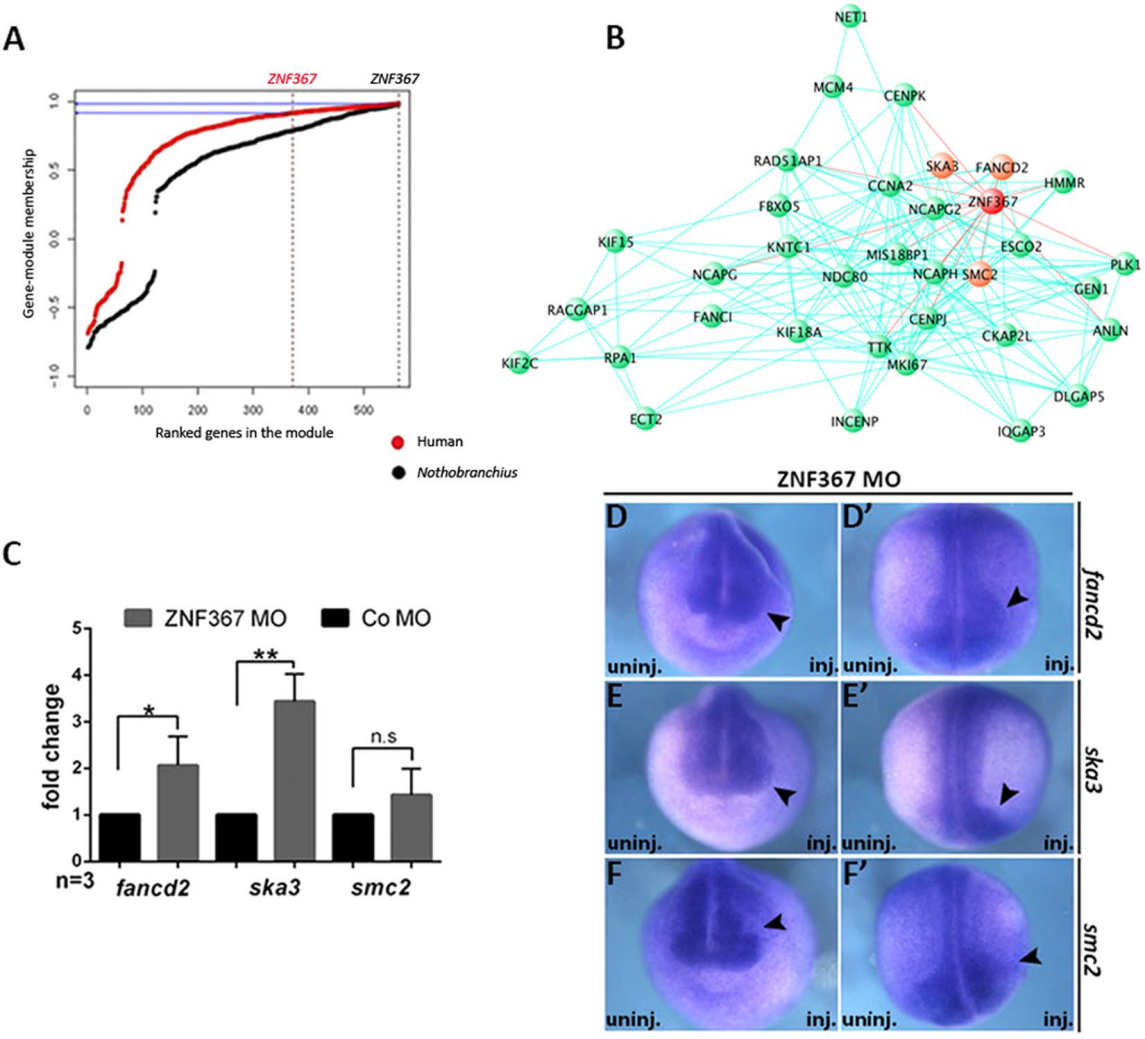
Network and molecular analysis of *znf367* neighbors. (**A**) Distribution of membership values for the analyzed module in human cells and *N*. *furzeri* brain. The vertical line indicates the rank of *znf367* and the horizontal line its membership value (black for *N*. *furzeri* and red for *H*. *sapiens*). Please note due to the high convexity of the human distribution *ZNF367* has very high membership despite its rank. **(B)** Central part of the gene module containing *ZNF367*. Only genes showing topological overlap >0.3 are shown. *ZNF367* is in red, while genes used for further analysis are in orange (*SMC2*, *SKA3* and *FANCD2*). **(C)** qRT-PCR analysis of *fancd2*, *ska3* and *smc2*. The levels of expression for the indicated mRNAs were evaluated for Co-MO or ZNF367-MO populations by qRT-PCR in triplicate and normalized to, *glyceraldehyde 3-phosphate dehydrogenase* (*gapdh*) expression. **(D)** mRNA distribution of *fancd2*, *ska3* and *smc2* in *znf367* morphants. Neurula stage embryos are shown in frontal (**D**,**E**,**F**) and dorsal view (D’,E’,F’). Arrowheads indicated the expanded domain of the mRNA distribution of each gene in the injected side of the embryo. Abbreviations: n, number of independent experiments; Error bars indicate standard error of the means (s.e.m); n.s., not significant. *p ≤ 0,05; **p ≤ 0,01. P-value were calculated by Student’s T-test.

Our previous results suggested that *znf367* represents a hub in the control of gene expression in the *N*. *furzeri* brain^2^. In order to test the conservation of this co-regulation across species, we analyzed CORTECON^20^ a public dataset of RNA-seq during cortical differentiation of human embryonic stem cells (hESCs) using weighted-gene co-expression network analysis (WGCNA)^21^. WGCNA constructs co-expression networks based on topological criteria. It was shown to be more robust than simple correlation and it has become the method of choice for gene expression studies in the nervous system^22,23^. This method identifies gene modules that are sets of tightly co-expressed genes, within the modules hub genes are identified as the genes with highest number of connections (highest connectivity) and are the putative drivers of the coherent expression of the genes within the module^21^. We therefore tested the conservation of gene co-expression networks between *N*. *furzeri* brain and human neuronal differentiation *in vitro* (Fig. 6A). We identified a conserved module that contains *znf367* as a hub gene and then tested whether *znf367* can be considered a hub in both species by computing its connectivity. *Znf367* was among the top connected genes in the gene module in both species (98% percentile in *N*. *furzeri* and 92% percentile in human cells). Gene Ontology overrepresentation analysis revealed that cell-cycle related terms are highly overrepresented in this module. It should be also noted that all these genes have high expression in the hESCs, they are down-regulated during early differentiation, and show a peak of expression around 12 days of differentiation *in vitro* that correspond to the period of cortical specification^20^. Among the genes that showed the highest topological overlap, we noted enrichment in genes known to be involved in the progression of mitosis and in the mitotic spindle checkpoint (Fig. 6B). This corroborates the idea that *znf367* has a role in the control of cell cycle and it could be preeminent in mitosis, when the dividing cell has the fundamental task of correctly arranging the genetic content of the two daughter cells. To verify our hypothesis, we decided to test by qRT-PCR and by whole mount *in situ* hybridization the expression of three of the genes closest to *znf367* in the network (Fig. 6B). We analyzed the expression level of *smc2*, *ska3* and *fancd2* in *znf367* morphants. The *smc2* gene codes for one of the condensin components of the Structural Maintenance of Chromosomes (SMC) protein complexes, which play key roles in the regulation of higher-order chromosome organization and its role is crucial in the chromatin compaction in prophase^24,25^. *Ska3* is one of the spindle and kinetochore-associated (Ska) proteins required for accurate chromosome segregation during mitosis^26^. During mitosis the cyclin-dependent kinase Cdk1 phosphorylates *SKA3* to promote its direct binding to the Ndc80 complex, (also present in the Znf367 network). This event is required for the overcoming of spindle checkpoint and the beginning of anaphase^26,27^. *FANCD2* encodes for a nuclear effector protein that is monoubiquitinated in response to DNA damage, targeting it to nuclear foci where it preserves chromosomal integrity^28^. Mutations in the FANC family are causative of the Fanconi Anemia in humans^29^. Greater than 60% of Fanconi anemia patients have developmental defects, such as growth retardation, short stature, microcephaly, and microphthalmia at birth, in addition to a highly elevated risk of bone marrow failure in the first decade of life^30^. This gene draws our attention as its knockdown in zebrafish embryos induced microcephaly, microphthalmia and pericardial edema^28^. It has been demonstrated that this factor is crucial for the S-phase rescue of damaged DNA, but also for the safeguarding of chromosome stability during mitosis^31,32^. The results obtained in three independent experiments, showed a significant increase in *fancd2* and *ska3* gene expression in *znf367* morphants. The *smc2* gene expression level followed the same trend without statistical significance (Fig. 6C). These results were also confirmed by WISH experiments: the expression domains of *fancd2 (*50%, N = 50), *ska3* (53%, N = 50) and *smc2* (48%, N = 50) were expanded on the ZNF367-MO injected side of the embryo (Fig. 6D–F’).

These suggest that *znf367* could have a major role in the control of chromosome stability and the functionality of the spindle check point.

## Discussion

We chose *Xenopus laevis* as a model system to directly modulate the *znf367* expression in the CNS without affecting other tissues and to unveil its role in tetrapods. *Znf367* is expressed in the neural tissue of the early *Xenopus laevis* embryo including the eye field and in the neural crest cells. The spatial expression pattern suggested a role in the context of primary neurogenesis. This was further supported by the marked loss of post mitotic neurons upon knockdown of *znf367*, suggesting that *znf367* could be essential for neuronal differentiation. In *Xenopus* znf367 morphants, we did not observe an increase in apoptosis rate suggesting that the loss of post-mitotic neurons was not due to unspecific morpholino toxicity or to a specific triggering of apoptotic pathways. Indeed, we found that the loss of *znf367* function led to an increased expression of genes involved in the maintenance of neuroblast and retinoblast as proliferating precursors. Accordingly, we observed a significant increase in the number mitotic cells in *znf367* morphants. The co-ordinate regulation of cell proliferation and differentiation is of fundamental importance in the development of the central nervous system^33^. At early developmental stages, a period of extensive proliferation is needed to generate the required number of progenitor cells for correct tissue and organ formation, accompanied or closely followed by differentiation^34^. After the closure of the neural tube, the epithelial lining of the ventricles becomes specialized, consisting of a single sheet of progenitor cells (neuroepithelial cells). These cells undergo symmetrical cell divisions during the proliferative period to self-renew and expand the pool of progenitors^34,35^. The subsequent transition from a proliferative neural precursor cell to a post-mitotic neurons is a highly regulated step, which, in many instances, has been shown to involve a cascade of transcription factors that is triggered by pro-neural genes^36^. Differentiation of neural progenitor cells requires withdrawal from the cell cycle, which is regulated by the expression of cell cycle inhibitors, such as *p27* in *Xenopus*^18^. Consistent with the increase in mitotically active cells, a reduced *p27* expression was observed in *znf367* morphants, thus raising the possibility that the neural progenitors are prevented from undergoing differentiation because they are not able to exit the cell cycle, remaining in an undifferentiated state. *Znf367* morphants also expressed high levels of *cyclin B1*, which is required to drive cells into mitotic division, but that must be degraded to allow anaphase. Again, this datum corroborates our hypothesis that *znf367* deficient neuroblasts could enter M phase, but they could not correctly exit mitosis and differentiate. Given the requirement of *znf367* for both proliferation and neuronal differentiation of neuroectodermal cells, it is plausible that *znf367* could be required to exit M phase or in the control of the spindle checkpoint that precedes anaphase. To have a wider view on the molecular mechanisms potentially regulated by *znf367*, we performed a correlation-based network analysis testing the conservation of gene co-expression networks between *N*. *furzeri* brain and human neuronal differentiation *in vitro*, identifying a conserved module that contains *znf367*. To this purpose we analysed the public RNA-seq database CORTECON. CORTECON reports transcript expression levels for all stages of *in vitro* differentiation from hESCs to cortical neurons, therefore capturing even the earliest stages of neurogenesis comparable to the stages analysed here in *Xenopus*.

We noted enrichment in genes involved in the regulation of the cell cycle and specifically in the progression of mitosis and mitotic spindle check point. The involvement of *znf367* in the control of cell cycle is supported by functional studies that demonstrated its implication in regulating different aspects of cancer progression^10^. Some of the genes, correlated to *znf367* in our correlation analysis are both implicated in CNS development as well as in cancer initiation and/or progression. Among these, we decided to closely analyze the relation between *znf367* and *smc2*, *ska3* and *fancd2*. SMC2 is part of the condensing complex required for the structural and functional organization of chromosomes^37^. Its role is crucial in the chromatin compaction in the prophase^24^. In our functional study the loss of *znf367* seemed to interfere with *smc2* mRNA level, but even if *smc2* mRNA seemed to be more abundant in *znf367* morphants than in controls, the results are suggestive of a trend, but not statistically significant. *Fancd2* is essential during zebrafish CNS development to prevent neural cell apoptosis during neuroblast proliferative expansion^28^. *FANCD2*, when mutated, is one of the causative genes of Fanconi anemia, an inherited disorder characterized by developmental defects, progressive bone marrow failure, and predisposition to cancer^30^. In particular, *FANCD2* in postnatal and adult life is required for the functional maintenance of the hematopoietic stem cell pool^38^. The link between *znf367* and *fancd2* seems therefore particularly intriguing since the *znf367* function seemed to be required to repress *fancd2* expression and allow cells to inactivate the spindle checkpoint and proceed towards differentiation. The level of *fancd2* mRNA is significantly up regulated in *znf367* morphants. It is tempting to speculate that during primary neurogenesis in *Xenopus znf367* could regulate *fancd2* expression levels in order to define the pool of neuroblast and coordinate the cell cycle exit necessary for post-mitotic differentiation. SKA3 is one of the spindle assembly checkpoint proteins. SKA3 is strongly associated with kinetochores during prometaphase and metaphase, while being diminished during anaphase and lost in telophase^27^. Its major role is to contribute to the silencing of spindle checkpoint during metaphase and to the maintenance of chromosome cohesion in mitosis^27,39^. In our *znf367* morphants, *ska3* is upregulated, supporting the idea that *znf367* could play a key role in the control of mitosis and in particular during metaphase. As *ska3* has to be downregulated to allow the progression towards anaphase and telophase. A possible hypothesis we can formulate is that the loss function of *znf367* could maintain abnormally high levels of *ska3*, keeping cells blocked in mitosis (metaphase). This analysis provided us with a deeper view of the possible action of *znf367* during neurogenesis but deeper analysis, necessary to shed light on the direct targets of znf367 and their function, should be addressed in the future.

In conclusion, we unveiled a role for *znf367* during neurogenesis in vertebrates. In particular, *znf367* emerged as a key controller of the neuroblast cell cycle, and it seemed to act regulating the events that are strictly controlled during the metaphase to allow the progression of the cell cycle and the onset of anaphase. The observed age-related down-regulation of *znf36*7 correlated with the age-related decline of quiescent aNSC to activate and give rise to new neural progenitors^2,12^. Our data shed light on the role of *znf36*7 during neural progenitors development giving a proof of concept of the continuity of molecular control in developing and adult neurogenesis. It will be of interest for future studies to identify both the upstream regulators and the downstream effectors of *znf367*. This is important not only due to the requirement of *znf367* during *X*. *laevis* neurogenesis, but more generally for the identification of the molecular factors involved in neuronal progenitors cell cycle exit and differentiation. If our findings will be validated also in adult neurogenesis they could represent the first step in defining new strategies to increase adult neurogenesis, leading to improved maintenance of functional aNSC.

## Methods

### Synteny analysis of znf367

Synteny analysis was performed using the NCBI GeneBank for the following organisms: *Xenopus laevis znf367*a (NP_001085362.1); *Xenopus laevis znf367b* (XP_018114684 PREDICTED); *Homo sapiens ZFF29A* (AY554164.1) and *Homo sapiens ZFF29b* (AY554165.1); *Nothobranchius furzeri* (HADY01011608.1).

### Embryo preparation

Animal handling and care were performed in strict compliance with protocols approved by Italian Ministry of Public Health and of the local Ethical Committee of University of Pisa (authorization n.99/2012-A, 19.04.2012). *Xenopus laevis* embryos were obtained by hormone-induced laying and *in vitro* fertilization then reared in 0.1 X Marc’s Modified Ringer’s Solution (MMR 1×: 0.1 M NaCl, 2 mM KCl, 1 mM MgCl_2_, 5 mM HEPES pH 7.5) till the desired stage according to Nieuwkoop and Faber^40^.

### Morpholino oligonucleotides, cloning and microinjections

ZNF367 antisense Morpholino oligonucleotides (MO) and a standard Control MO were provided by Gene Tools, Philomath, OR, USA. ZNF367 MO sequence: 5′-CAGCCTATCTGACATTTGTTACTAC-3′. Co MO sequence: 5′-CCTCTTACCTCAGTTACAATTTATA-3′. Microinjections were performed as described previously^41^. Injected MO amounts were: 9 ng ZNF367 MO and 9 ng Control MO. Correct injections were verified by co-injected of 250 pg of GFP mRNA and using a fluorescence microscope. The un-injected side represents an internal control in each embryo. For functional rescue experiments, the open reading frame of *X*. *laevis znf367* (XM_018259195.1 PREDICTED) was cloned into the pCS2+. For Rescue experiments, 9 ng ZNF367 MO and 500 pg full-length *znf367* mRNA were co-injected. Higher doses of morpholino, 12 or 15 ng per embryos, were tested. Morphants injected with 12 or 15 ng of morpholino showed the described phenotype of altered neurogenis but also defects in the neural tube closure that were not efficiently rescued by the injection of znf367 mRNA (not shown). For this reason all the experiments showed in the results section were performed at the 9 ng ZNF367 MO dose. Capped *znf367* mRNA was obtained using the MegaScript *in vitro* transcription kit (Ambion), according to manufacturer’s instructions.

### *In situ* hybridization (ISH) experiments

Whole mount *in situ* hybridization (WISH) approaches was performed as described^42^. BM purple (Roche) was used as a substrate for the alkaline phosphatase; digoxigenin-11-UTP-labelled sense, and antisense RNA probes were generated via *in vitro* transcription. After color development, embryos were post-fixed and bleached over light to remove the pigment. For ISH on cryosections (12 µm), embryos were fixed in 4% paraformaldehyde (PFA) in PBS, cryoprotected with 30% sucrose in PBS and embedded in Tissue-Tek O.C.T. compound (Sakura, 4583). ISH on cryosections was performed as described^42^. Unpublished new plasmids for *in situ* hybridization were generated as follows: *X*. *laevis* znf367 EST clone image (ID_6637026) was cloned in pBKS-vector. *X*. *laevis*-Ska3 (NM_001127749), fancd2 (NM_AY633665) and smc2 (NM_001087904) were obtained by RT-PCR and cloned in pGEM-T vector. The following plasmids were used for preparation of antisense RNA probes, enzyme used for linearization and polymerases are indicated: *X*. *laevis* znf367 EST-pBKS- (XhoI, T7); pcna-pBSK (SalI, T7); sox2-pCS2+ (EcoR1, T7); n-tubulin-pBKS (NotI, T3); elrC-pBKS (NOTI, T7); elrD-pBSK (XhoI, T3); rx1^42^. nrg1-pBKS (BamHI, T3); p27-Pbsk (BamHI, T7). *X*. *laevis* fancd2-pGEM-T (ClaI; SP6); *X*. *laevis* ska3-pGEM-T (NcoI; SP6); *X*. *laevis* smc2-pGEM-T (NcoI; SP6).

### TUNEL and PH3 staining in *Xenopus*

TUNEL (TdT-mediated dUTP-dig nick end labeling) and PH3 (phosho histone 3) staining was performed at neurula stage according to established protocols^41,43^. TUNEL and PH3 positive cells were counted within defined areas in control and injected side of each manipulated embryo. P-values were calculated by paired Student’s T-test using GraphPad Prism 6 software (San Diego, CA, USA). Statistical significance was indicated as: *p ≤ 0.05, **p ≤ 0.01, ***p ≤ 0.001, ****p ≤ 0.0001.

### Quantitative Reverse Transcription Polymerase Chain Reaction (qRT-PCR)

Total RNA was extracted from 30 *Xenopus* morphants (at stage 18) using Nucleospin® RNA (Macherey-Nagel) according to the manufacturer’s instruction. cDNA was prepared by using iScript™ cDNA Synthesis Kit (Bio-Rad) and quantitative real-time PCR was performed using GoTaq®qPCR master mix (Promega) according to the manufacturer’s instruction. Relative expression levels of each gene were calculated using the 2^−ΔΔCt^ method^44^ and normalized to glyceraldehyde 3-phosphate dehydrogenase (GAPDH). The following primers were used to perform qRT-PCR: *pcna*^45^ (Forward: 5′-CGTCGCGGTAATCCCTTA-3′; Reverse: 5′-TTGACCTCCTAGGGCAGAGA-3′); *N-tubulin* and *sox2* (De Robertis’ lab, web site: http://www.hhmi.ucla.edu/derobertis/); *elrC*^46^ (Forward: 5′-GCTTTCTATCCTCCCCAGGT-3′; Reverse: 5′-TGCCACAGGACACTCTCATC-3′); *cby1* (Forward: 5′-TGAAGCGGTTCCAGTTGTCG-3′; Reverse: 5′-TTGGTGGCAACAACCCTCTT-3′); *ska3* (Forward: 5′-ACCGGAACTTTCCTACAGGC-3′; Reverse: 5′-ATTTCTGGGCGTGTTGGTGT-3′); *fancd2* (Forward: 5′-CCCTACACTCACCAGGCAAAC-3′; Reverse: 5′-AGCGTTTCAGCTTTCTTGCTATT-3′); *scm2* (Forward: 5′-GCTGAAAGAGAGAAGAAACGCAAA; Reverse: 5′-CTTGCAGAGAGCTCAGACCATC-3′); *rx1* (Forward: 5′-GAGGAACCGGACAACATTCAC-3′; Reverse: 5′-TCATAGCCAGCTCTTZCTCTGC-3′); *gapdh* (Forward: 5′-CTTTGATGCTGATGCTGGA-3′; Reverse: 5′-GAAGAGGGGTTGACAGGTGA-3′).

### Statistics

Statistical analysis for qRT-PCR experiments were performed by Student’s T-test using GraphPad Prism 6 software (San Diego, CA, USA). The levels of mRNA expression for individual genes were evaluated for Control-Morpholino or ZNF367-Morpholino injected embryos by qRT-PCR. The results obtained in three independent experiments were normalized to the expression of the hosekeeping gene, *gapdh*. The mean of the Control-Morpholino was set 1. The relative mRNA levels were calculated using the comparative C_t_ method (2^−ΔΔCt^)^44,47^. Statistical significance was indicated as: *p ≤ 0.05.

Statistical analysis for the phenotypes observed after the injection of the Control-Morpholino or the injection of ZNF367-Morpholino, were performed by Student’s T-test using GraphPad Prism 6 software (San Diego, CA, USA). We compared the percentage of the embryos with altered markers gene expression between the Control-Morpholino injected embryos and the ZNF367-Morpholino injected embryos. Statistical significance was indicated as: **p ≤ 0.01, ***p ≤ 0.001, ****p ≤ 0.0001.

### WGCNA (Weighted Gene Co-expression Network Analysis)

Network analysis was performed using WGCNA method^21^. Samples used for the workflow were derived from two independent datasets, one from *Nothobranchius furzeri*’s brain, comprehensive of two strains (MZM-04010 and GRZ), six different time points and five replicates per time point^2^, and the other one from human embryonic stem cells. In particular the latter was obtained from a cerebro-cortical developmental experiment performed on hESC with 9 different time points^20^.

Network analysis was performed through different steps:

- Setting of the soft threshold, coefficient necessary for the adjacency matrix construction, as shown in the formula:
  $${a}_{{\bf{i}}{\bf{j}}}={|cor({x}_{{\rm{i}}},{x}_{{\rm{j}}})|}^{{\rm{\beta }}}$$
- Adjacency matrix and TOM (Topological Overlap Matrix), defined as:
  $$TO{M}_{ij}=(\sum {a}_{iu}\,\ast \,{a}_{uj}+{a}_{ij})/{\rm{\min }}({k}_{i},{k}_{j})+1-{a}_{ij}$$
- Hierarchical clustering and modules detection after measuring the module eigengenes; every module is characterized by a color (as the module which has been studied for the analysis, defined by the turquoise color)
- Module-trait relationship table construction, as correlation between single gene expression and external trait (in this case aging/development)
- Module membership plot (as correlation between single gene expression and module eigengene): this was done for both the *N*. *furzeri* and the *H*. *sapiens* datasets, as described in Fig. 6A
- Visualization with Cytoscape software.

Network construction was done in two independent analyses: the first one only on *Nothobranchius furzeri* dataset, while the latter using a consensus network obtained matching the two datasets. As soft threshold, we chose β = 6 for both the analyses to obtain the correspondent adiacency matrix and TOM, and significant modules negatively correlated with *N*. *furzeri* brain aging were selected. The genes contained in the selected modules were then tested for GO analysis using WebGestalt software, and then visualized using Cytoscape. Finally, the overall module membership of the genes contained in the “turquoise” module (as specified above, and only for the second analysis) was plotted on the ranked genes for both the killifish and the human data.

### Data availability

All data generated or analysed during this study are included in the manuscript.
